# Letter to the Editor: Response to “Diagnostic yield, safety and therapeutic consequences of myocardial biopsy in clinically suspected fulminant myocarditis unweanable from mechanical circulatory support”

**DOI:** 10.1186/s13613-023-01232-8

**Published:** 2024-01-09

**Authors:** A. S. Giordani, A. Baritussio, R. Marcolongo, A. L. P. Caforio

**Affiliations:** Cardiology, Department of Cardiac Thoracic Vascular Sciences and Public Health, University of Padua, Via Giustiniani 2, 35128 Padua, Italy

We read with great interest the article by Marquet et al. [1]. The Authors should be praised for focusing on the clinical relevance of performing myocardial biopsy (MB) in cardiogenic shock (CS) requiring mechanical circulatory support (MCS). This single-center retrospective cohort consisted of 47 clinically suspected fulminant myocarditis (FM) patients on MCS undergoing MB: 30 MBs were surgical (64%) and 17 were endomyocardial (EMB, 36%). A sizeable rate of complications is shown, in particular pericardial tamponade (29% after EMB, 10% after surgical MB), and one death. As expected, after MB etiological diagnosis rate significantly increased (from 47% pre-MB to 62% post-MB, *p* = 0.02), and consistently, therapeutic modifications were performed more frequently (19–32%, *p* = 0.03). Nevertheless, the Authors outline that the rate of “alternate diagnoses” (30–32%) and of “therapeutic modifications leading to recovery” (4–8%) were non-significantly impacted by MB execution.

First, we would like to outline that the study results may have been influenced by the peculiar selection criteria. Patients were included only if MB was performed at the time of MCS implantation: this may imply that EMB was performed too late to reveal an alternative diagnosis than FM and to have a significant impact on the disease course. Indeed, current guidelines [2] and a recent Consensus Statement by the three major International Heart Failure Societies [3] recommend considering MB for all clinically suspected myocarditis cases, irrespective of hemodynamic stability. Similarly to other diagnostic tools, if MB is performed too late, it may not be as beneficial as it could potentially be. It would be useful to know the time span between hospital admission and MCS, to identify what the best moment for EMB could have been, since time–performance relationship is key for the results’ interpretation.

Second, with respect to the possible alternative diagnosis to FM, if other possible causes of CS have been excluded through a guideline-based approach [2] (coronary artery disease was excluded in only 66%, and one MB showed myocardial infarction), myocarditis evidently remains likely, since it causes up to 15% of non-ischemic CS cases [4]. But importantly, even if acute myocarditis already is the most likely diagnosis, MB is necessary to

- achieve diagnosis of certainty, especially in critical settings in which it may be difficult to exclude phenocopies. This is even more important if non-invasive tests are unavailable: only 9 patients underwent cardiac magnetic resonance (CMR), which was diagnostic in only 6 cases; 3 cases would have been missed by omitting MB. Histological diagnosis can potentially guide treatment, although robust data on the use of IT in clinically suspected or biopsy proven myocarditis patients under MCS are currently lacking.
- exclude the presence of infectious agents in the myocardium, since immunosuppressive therapy (IT) should not be used in viral myocarditis [2]. Polymerase chain reaction (PCR) is the only validated method to exclude myocardial infection, and viral serology has no relevance for viral myocarditis diagnosis [5]; remarkably, no robust evidence supports the Authors stating that “most viral myocarditis can be proven with noninvasive testing”.
- achieve an etiological diagnosis, which is essential to guide IT: some peculiar types of myocarditis, i.e., eosinophilic and giant cell myocarditis (GCM), typically present as FM and deserve specific treatment. It is noteworthy that achieving GCM diagnosis before heart transplant referral may be important, since GCM can even relapse in the transplanted heart in a minor, yet relevant, percentage of cases (8% according to a recent meta-analysis [6]).

Remarkably, 38% of MB did not lead to diagnosis in this study: this raises concern on the reliability of the Bonaca criteria for myocarditis diagnosis, in comparison with internationally validated criteria [2, 3].

Third, the rate of changes in “therapeutic modifications leading to recovery” may have been influenced by the fact that IT for autoimmune virus-negative myocarditis could have been halted by absolute contraindications, such as active infections, liver, or renal failure (30% of patients were on renal replacement therapy at the time of MB). Moreover, the efficacy of an etiology-directed treatment may have been reduced by a delayed onset. Furthermore, even if not *statistically* significant, MB led to a decisive treatment redefinition in 2 cases; in a potentially fatal condition such as FM, especially in case of younger patients (median patients’ age was 41), every single success seems clinically and ethically relevant. In these dramatic scenarios, the use of the gold standard technique is essential.

Finally, the rate of complications shown in the present series is higher than previously reported [3], mainly represented by pericardial tamponade. This may be explained by the high rate of anticoagulation (87%), as required for most MCS types, before MB. Notably, the rate of non-fatal complications was still inferior to the 55% death rate observed in this cohort, which could lead to carefully reconsider the risk/benefit ratio of MB in critical cases.

In conclusion, MB, the myocarditis diagnostic gold standard, should not be restricted to unweanable MCS patients, but should be used as soon as possible, to maximize its diagnostic yield and provide a rapid etiological diagnosis to drive treatment. We hope this study paves the way forward for improving EMB integration in FM diagnostic workflow, prioritizing its essential role.

## Data Availability

Not applicable.

## Abbreviations

CMR: Cardiac magnetic resonance
CS: Cardiogenic shock
EMB: Endomyocardial biopsy
GCM: Giant cell myocarditis
IT: Immunosuppressive therapy
MB: Myocardial biopsy
PCR: Polymerase chain reaction
